# Soil gross nitrogen transformations in forestland and cropland of Regosols

**DOI:** 10.1038/s41598-020-80395-x

**Published:** 2021-01-08

**Authors:** Xiao Ren, Jinbo Zhang, Hamidou Bah, Christoph Müller, Zucong Cai, Bo Zhu

**Affiliations:** 1 Key Laboratory of Mountain Surface Processes and Ecological Regulation, Institute of Mountain Hazards and Environment, Chinese Academy of Sciences, #9, Block 4, Renminnanlu Road, Chengdu, 610041 Sichuan China; 2 University of Chinese Academy of Sciences, Beijing, 100049 China; 3 School of Geography Sciences, Nanjing Normal University, Nanjing, 210023 China; 4 Institute of Plant Ecology, Justus-Liebig University Giessen, Heinrich-Buff-Ring 26, 35392 Giessen, Germany; 5 School of Biology and Environmental Science and Earth Institute, University College Dublin, Belfield, Dublin, Ireland

**Keywords:** Biogeochemistry, Environmental sciences

## Abstract

Soil gross nitrogen (N) transformations could be influenced by land use change, however, the differences in inherent N transformations between different land use soils are still not well understood under subtropical conditions. In this study, an ^15^N tracing experiment was applied to determine the influence of land uses on gross N transformations in Regosols, widely distributed soils in Southwest China. Soil samples were taken from the dominant land use types of forestland and cropland. In the cropland soils, the gross autotrophic nitrification rates (mean 14.54 ± 1.66 mg N kg^−1^ day^−1^) were significantly higher, while the gross NH_4_^+^ immobilization rates (mean 0.34 ± 0.10 mg N kg^−1^ day^−1^) were significantly lower than those in the forestland soils (mean 1.99 ± 0.56 and 6.67 ± 0.74 mg N kg^−1^ day^−1^, respectively). The gross NO_3_^−^ immobilization and dissimilatory NO_3_^−^ reduction to NH_4_^+^ (DNRA) rates were not significantly different between the forestland and cropland soils. In comparison to the forestland soils (mean 0.51 ± 0.24), the cropland soils had significantly lower NO_3_^−^ retention capacities (mean 0.01 ± 0.01), indicating that the potential N losses in the cropland soils were higher. The correlation analysis demonstrated that soil gross autotrophic nitrification rate was negatively and gross NH_4_^+^ immobilization rate was positively related to the SOC content and C/N ratio. Therefore, effective measures should be taken to increase soil SOC content and C/N ratio to enhance soil N immobilization ability and NO_3_^−^ retention capacity and thus reduce NO_3_^−^ losses from the Regosols.

## Introduction

Nitrogen (N) is an essential element for plant growth, and its form and amount are mainly controlled by N production and consumption processes in natural soils^1–3^. In terrestrial ecosystems, if N cannot be efficiently conserved in soils, potential N losses will induce negative effects on the climate, the environment and even human health^4–6^. Therefore, it is essential to quantify the simultaneously occurring N production and consumption processes to identify whether soils can effectively conserve N.

Soil gross N transformations, driven by microorganisms, are greatly impacted by many soil properties and climatic conditions, such as soil temperature and moisture, soil pH, substrate concentration (NO_3_^−^, NH_4_^+^ and organic N), and the quality and quantity of organic materials^7–11^. For instance, soil pH affects microbial community composition and activities, and is therefore important for NO_3_^−^ production and consumption processes^7,12,13^. Soil carbon (C) and N availability can influence gross mineralization and NH_4_^+^ immobilization processes, which are important for assessing the indigenous soil N supply^14,15^. In general, the above mentioned factors are significantly affected by different land uses^16–18^, which thus induce differences in soil N transformations^10,19,20^.

With the development of ^15^N dilution technique and numerical model methods, studies on the gross N transformations in different land uses have been greatly increasing^4,9,10,20,21^. Generally, previous studies indicated that in natural forest ecosystems, most available N could be effectively conserved in soils through inherent soil N conservation mechanisms^6,14^. For instance, in subtropical forest soils, low gross mineralization rates combined with negligible gross nitrification rates were confirmed as an effective N conservation mechanism through reducing the production of inorganic N^9^. Furthermore, Zhang et al. found that the coexistence of high inorganic N production and immobilization rates was also an effective N conservation mechanism in subtropical forest soils^4^. However, after forestland conversion to cropland, the changes of vegetation cover and management practices (e.g. tillage and fertilization) could significantly influence soil N transformations^16,17,19,20^. In subtropical acid soils of Southwest China, Xu and Xu reported that compared to forestland soil, the cropland soils had significantly higher gross nitrification rates and lower immobilization rates, which resulted more NO_3_^−^ losses through leaching or denitrification^9^. However, the mechanisms of different land uses influencing N transformation processes may be quite different according to soil types in subtropical regions. Therefore, more detailed studies are still needed to quantify the inherent N transformation processes under different land use soils in other important soil types in subtropical regions, which is beneficial for understanding whether those different soils can effectively conserve N.

The Sichuan Basin in Southwest China is a subtropical region characterized by numerous hills. Regosols (locally known as purple soil) are the most important and widely distributed cropland soils in this region, covering an area of more than 1.6 × 10^5^ km^2^. Unlike the normally occurring soils in subtropical regions, Regosols are weakly developed mineral soils and are characterized by a shallow soil layer, coarse-textured sandy loams and good soil aeration^22^. Consequently, Regosols are susceptible to erosion and leaching, and the large N losses via leaching and overland runoff may cause local and widespread non-point source N pollution^23^. A previous study reported that annual nitrate leaching losses from sloping croplands could be up to 53.4 kg N ha^−1^ year^−1^ in this region, which was the dominant N loss pathway^24^. Mitigating this high loss rate needs to enhance the soil N retention capacity by understanding inherent soil N cycling mechanisms in Regosols. Many studies have indicated that soil N transformations predominantly regulate N forms and composition in soils^14,25^. In particular, Zhang et al. revealed that the NO_3_^−^ proportion was mainly regulated by the process of nitrification^26^. However, the inherent soil N transformation processes are still not well understood in the Regosols of the Sichuan Basin. Wang et al. investigated the soil gross N transformations in cropland soils under different fertilization regimes in this region and found that the increased gross nitrification rates governed the increases in cumulative NO_3_^−^ losses via interflow and overland runoff^18^. In the Sichuan Basin, the dominated land uses are forestland and sloping cropland. The difference in land uses can cause significant differences in soil properties^27^, which thus may affect soil gross N transformations. However, to date, how different land use soils influence gross N transformations in Regosols is not fully understood in this region. Understanding N transformations in Regosols and the effects of different land uses would provide important information for assessing the risks of N losses, and further provide the scientific basis for how to regulate the N transformation process to mitigate N losses in the Sichuan Basin of Southwest China.

In this study, we quantified gross N transformations in forestland and cropland of Regosols in the Sichuan Basin of Southwest China. This study aimed to (1) investigate the characteristics of gross N transformations in Regosols, (2) examine the differences in soil N transformations for different land uses in Regosols, and (3) evaluate the N conservation potential and N loss risks of Regosols under different land uses.

## Materials and methods

### Study region

This experiment was conducted at the Yanting Agro-Ecological Station of Purple Soil, Chinese Academy of Sciences, Yanting County of Sichuan Province (31° 16′ N, 105° 27′ E)^23^. It situates in the central Sichuan Basin and exhibits a moderate subtropical monsoon climate, with an average annual temperature and rainfall of 17.2 °C and 836 mm (30-year mean), respectively. The soil, classified as Eutric Regosols by the FAO soil classification^28^, is locally termed “purple soil” due to its purplish colour^23^. It is typically non-zonal and weakly developed mineral soil, mostly characterizing by a neutral or alkaline reaction^18,23^. Currently, forestland and sloping cropland are the main land uses in this area.

### Site description

To investigate the differences of inherent N transformations between different land use soils, sampling was conducted from the two main land uses of forestland and cropland. The forestland was initially planted with *Alnus cremastogyne* and *Cupressus funebris* in the 1970s to reforest cropland, and then, this site experienced natural succession without artificial management. It is now dominated by the representative forest type of *Cupressus funebris* in the study region, with a density of 1595 stems ha^−1^. The selected forestland site was with an area of approximately 1.3 ha^−1^. The cropland site was adjacent to the forestland site, with an area of 100 m × 100 m. It has been cultivated for more than 50 years, conventionally rotated with winter wheat (*Triticum aestivum* L.) and summer maize (*Zea mays* L.). Fertilizers in the winter wheat and summer maize seasons were applied at the same amounts of K (36 kg K_2_O ha^−1^) and P (90 kg P_2_O_5_ ha^−1^), but at different rates of N (130 and 150 kg N ha^−1^ as ammonium bicarbonate, respectively). All the fertilizers were manually applied and incorporated into the surface soil (0–20 cm) together with harrowing (approximately 20 cm deep). No irrigation was applied during either the wheat or maize season. The forestland and cropland sites had the same soil type (Regosols) and slope (5%).

### Field soil sampling and N loss monitoring

For forestland and cropland sites, grids with an area of 10 m × 10 m were uniformly divided. Then eleven and eight grids were randomly selected for soil sampling from different slope positions (i.e. upslope, middle slope, and downslope) in forestland and cropland sites, respectively, to minimize the potential effect of spatial heterogeneity on the experimental results. Furthermore, to minimize the effects of fertilization on gross N transformations in cropland soils, soil samples were collected in April 2016 when closed to the wheat harvest. At each sampling grid, the organic layer was removed first if present and three soil cores were randomly taken from the surface 0–20 cm layer. Then, they were well mixed, passed through a 2-mm sieve, and ultimately separated into two sub-samples. One sample was air-dried for soil property analyses, and the other sample was stored for the incubation experiment at 4 °C for < 1 week.

To better identify how soil N transformations regulate the mechanisms involved in NO_3_^−^ losses, field monitoring of NO_3_^−^ loss was conducted following each rainfall event during 2016. At each site, NO_3_^−^ concentration in surface soil (0–20 cm) was also continually monitored at least once a week. Taking the selected sloping forestland site as a whole drainage area (1.3 ha^−1^), the discharge was monitored by the triangle weir installed at the outlet and the water samples were taken from the collecting tanks installed under the weir. At the cropland site, lysimeters were permanently established to take both interflow and overland runoff water samples^23^. Following each runoff event, the discharges of both interflow and overland runoff were determined. For both forestland and cropland sites, water samples were collected using 500-mL polyethylene bottles for assaying NO_3_^−^ concentration. The annual NO_3_^−^ loss flux (*Q*, kg N ha^−1^ year^−1^) was estimated as follow:

1 $$Q = \mathop \sum \limits_{i = 1}^{n} \left( {C_{i} \times q_{i} /100} \right)$$

where *C*_*i*_ is the NO_3_^−^ concentration in the interflow and overland runoff sample (mg L^−1^), *q*_*i*_ is the interflow and overland runoff discharges (mm), and *n* is the number of runoff events during the monitoring period. During the experimental period, the daily precipitation was automatically monitored by a meteorological station located at a distance of approximately 3 km from the sampling sites.

### ^15^N tracing experiment and model

The inherent gross N transformations in the forestland and cropland soils were determined using a ^15^N tracing technique. Two ^15^N sources, ^15^NH_4_NO_3_ (9.44 atom% excess) and NH_4_^15^NO_3_ (9.75 atom% excess), were used in this study. For each soil sample (three replicates for each ^15^N labelling treatment), we prepared a series of conical flasks (250-mL) using 20 g of fresh soil (oven-dry basis) and added NH_4_^15^NO_3_ or ^15^NH_4_NO_3_ solutions to obtain the same NH_4_^+^-N and NO_3_^−^-N concentrations (20 mg N kg^−1^). Then they were adjusted to 60% WHC (water-holding capacity). In agricultural soils, the transformation of NH_4_^+^ to NO_3_^−^ was generally fast^6,21^. To avoid the low NH_4_^+^ concentrations in soils after incubation and guarantee the ^15^N detection requirements^29,30^, therefore, relatively high application amounts of NH_4_NO_3_ were added compared to initial NH_4_^+^ and NO_3_^−^ concentrations in this study. After 0.5, 12, 24, and 48 h of incubation at 25 °C, soils were extracted to measure the NH_4_^+^ and NO_3_^−^ concentrations and isotopic composition. Distillation with MgO and Devarda’s alloy were conducted to separate NH_4_^+^ and NO_3_^−^, strictly following the procedures described in previous studies^31,32^. Before separating, the recovery of NH_4_^+^ and NO_3_^−^ in a standard solution (1 g NH_4_^+^-N/NO_3_^−^-N L^−1^) was determined. The results showed that the recovery of NH_4_^+^-N and NO_3_^−^-N in the solution was more than 99% and 95%, respectively. Finally, the ^15^N abundances of NH_4_^+^ and NO_3_^−^ were analyzed by an automated C/N analyser and isotope ratio mass spectrometer (IRMS 20–22, SerCon, Crewe, UK).

**Figure 1:**
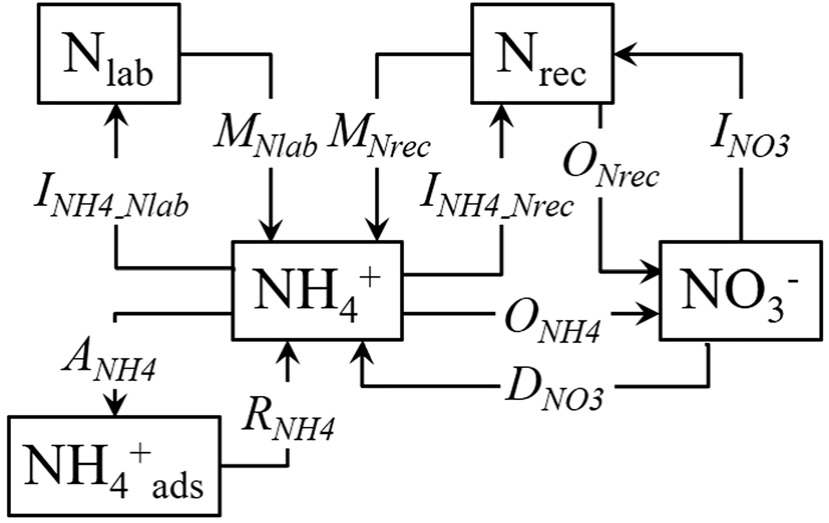
^15^N tracing model used for data analysis. (*N*_*lab*_ labile soil organic N, *N*_*rec*_ recalcitrant soil organic N, *NH*_*4*_^*+*^_*ads*_ adsorbed NH_4_^+^).

The widely used numerical ^15^N tracing model was employed to investigate the gross N transformations in this study. For the details of this model, numerous previous studies can be referenced^18,21,31,32^. Briefly, this model mainly involved the following ten simultaneously-occurring processes (Fig. 1): *M*_*Nlab*_ and *M*_*Nrec*_, mineralization of labile organic N and recalcitrant organic N to NH_4_^+^, respectively; *I*_*NH4-Nlab*_ and *I*_*NH4-Nrec*_, immobilization of NH_4_^+^ to labile organic N and recalcitrant organic N, respectively; *A*_*NH4*_ and *R*_*NH4*_, adsorption and release of adsorbed NH_4_^+^ on cation exchange sites, respectively; *O*_*NH4*_, oxidation of NH_4_^+^ to NO_3_^−^ (autotrophic nitrification); *O*_*Nrec*_, oxidation of recalcitrant organic N to NO_3_^−^ (heterotrophic nitrification); *D*_*NO3*_, dissimilatory NO_3_^−^ reduction to NH_4_^+^ (DNRA); and *I*_*NO3*_, immobilization of NO_3_^−^ to recalcitrant organic N^29,30^. The transformation rates were calculated by zero-order, first-order or Michaelis–Menten kinetics by minimizing misfits between the modelled and determined concentrations and ^15^N enrichments of NH_4_^+^ and NO_3_^−^ (averages ± standard deviations). Aikaike’s Information Criterion (AIC) was utilized to select the best model, and the Markov chain Monte Carlo-Metropolis algorithm (MCMC-MA) was employed for optimizing the parameter^21,30,32^. The MCMC-MA routine was conducted using MATLAB software (Version 7.2, The MathWorks Inc.). Finally, average transformation rates over a 48 h period (mg N kg^−1^ day^−1^) were calculated on the basis of the kinetic settings and the final parameters.

The total mineralization rates (*M*_*N*_) were estimated as the sum of *M*_*Nlab*_ and *M*_*Nrec*_, and the total NH_4_^+^ immobilization rates (*I*_*NH4*_) were calculated as the sum of *I*_*NH4-Nlab*_ and *I*_*NH4-Nrec*_. Nitrification capacity was expressed as the ratio of *O*_*NH4*_ to *M*_*N*_. The NO_3_^−^ retention capacity was defined as the ratio of NO_3_^−^ consumption (*I*_*NO3*_ + *D*_*NO3*_) to total nitrification (*O*_*NH4*_ + *O*_*Nrec*_).

### Soil chemical property measurements

Soil property analyses strictly followed the procedures described in Soil Agro-Chemical Analysis^33^. Soil pH was measured in a 1:2.5 (soil-to-water) suspension using a DMP-2 mV/pH detector (Quark Ltd, Nanjing, China). Soil organic carbon (SOC) and total N (TN) were measured by wet digestion with H_2_SO_4_–K_2_Cr_2_O_7_, and by semi-micro Kjeldahl digestion using Se, CuSO_4_ and K_2_SO_4_ as catalysts, respectively. The concentrations of NH_4_^+^ and NO_3_^−^ were measured by extraction with 2 M KCl, filtration with filter paper, and analysis using an AA3 continuous-flow analyser (Bran + Lubbe, Norderstedt, Germany).

### Statistical analyses

All statistical analyses were carried out in SPSS 20.0 software (SPSS Inc., Chicago, IL, USA). The Kolmogorov–Smirnov test was conducted first to test the normality of the data. The differences in the gross N transformation rates and soil properties between the cropland and forestland soils were detected using an independent sample *t* test. The significance level was conventionally set at 0.05. The relationships between gross N transformation rates and soil properties were tested by Pearson correlation analysis.

## Results

### Soil properties

**Figure 2:**
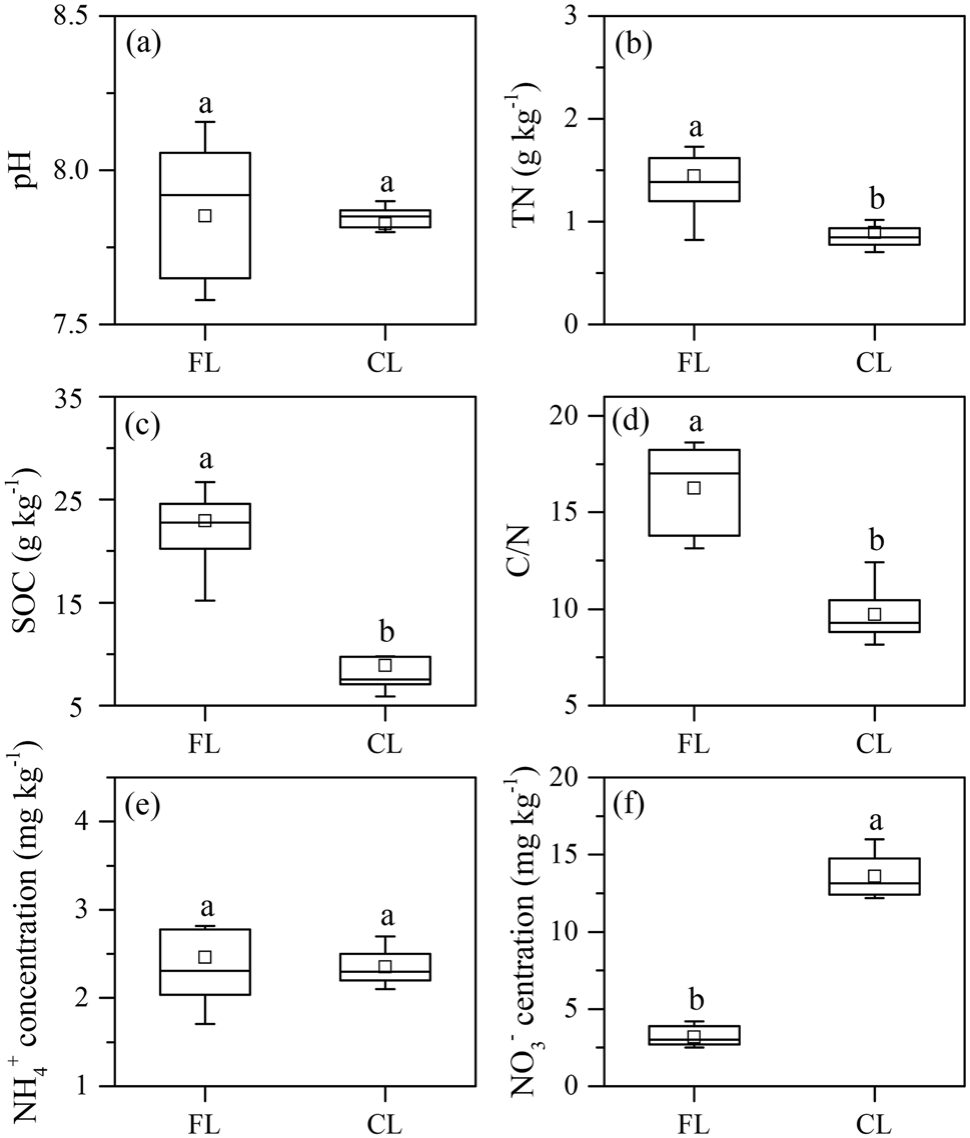
Soil properties of pH (**a**), TN (**b**), SOC (**c**), C/N (**d**), NH_4_^+^ concentration (**e**) and NO_3_^−^ concentration (**f**) in the forestland and cropland. *FL* forestland, *CL* cropland. The different letters in each sub-figure indicate significant differences between the different land use soils (*P* < 0.05). The bottom/top of the box denote 25th/75th percentiles. Whiskers denote 5th/95th percentiles. The squares denote the mean values, and the black lines denote the mid-values.

The average pH was 7.85 ± 0.06 and 7.83 ± 0.03 in the forestland and cropland soils, respectively, with no significant difference (Fig. 2a). However, the average SOC content (22.91 ± 1.37 g kg^−1^), TN content (1.45 ± 0.13 g kg^−1^) and C/N ratio (16.26 ± 0.65) in the forestland soils were significantly greater than those in the cropland soils (mean 8.90 ± 1.21 g kg^−1^, 0.90 ± 0.07 g kg^−1^ and 9.72 ± 0.51 for SOC, TN and C/N, respectively, *P* < 0.05) (Fig. 2b–d). The average soil NH_4_^+^ concentrations were 2.46 ± 0.18 mg N kg^−1^ and 2.35 ± 0.07 mg N kg^−1^ in the cropland and forestland soils, respectively, with no significant difference (Fig. 2e). The average soil NO_3_^−^ concentration in the cropland soils (13.60 ± 0.51 mg N kg^−1^) was significantly greater (*P* < 0.05) than that in the forestland soils (3.02 ± 0.08 mg N kg^−1^) (Fig. 2f).

### Soil gross N transformation rates

**Figure 3:**
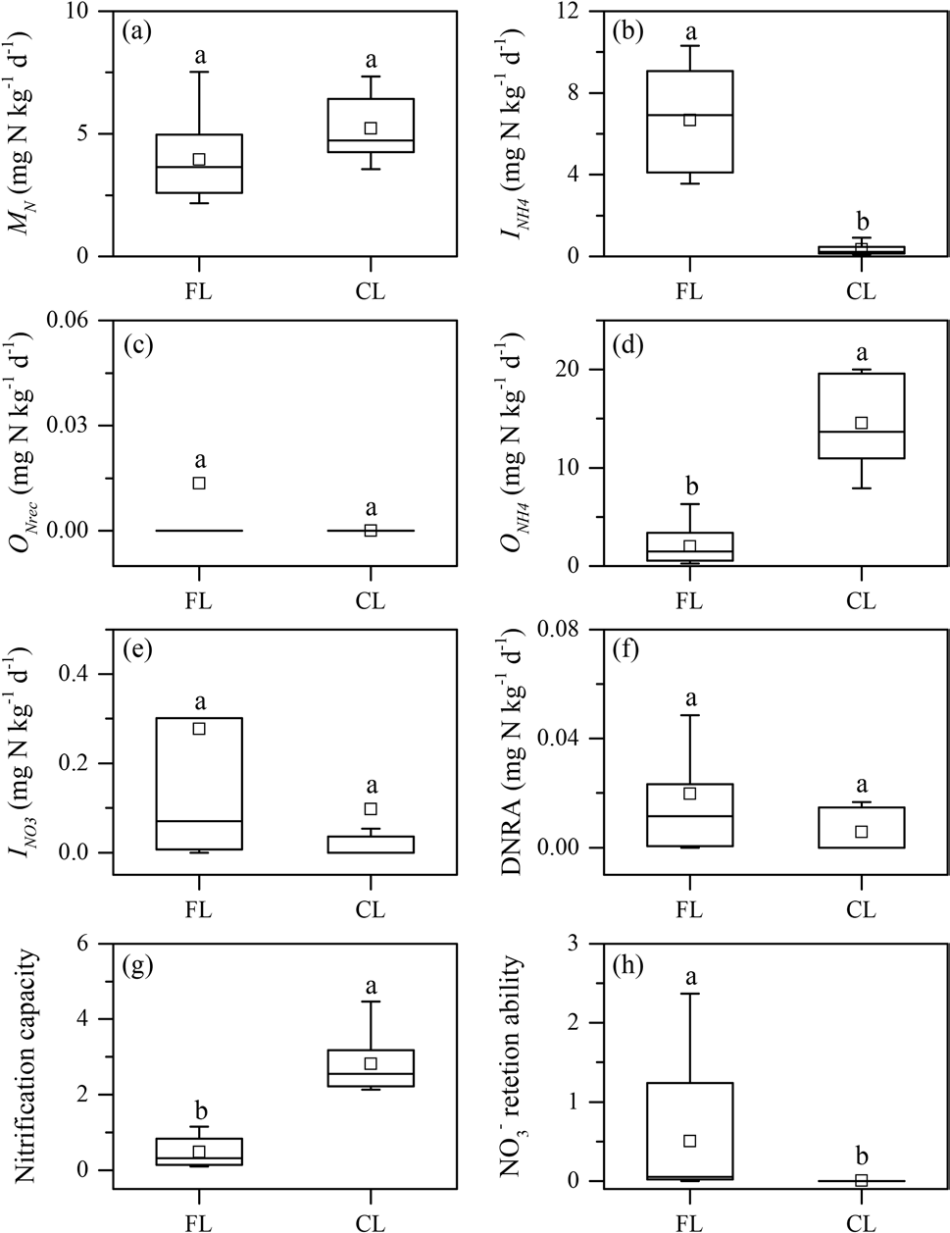
Gross N transformation rates in the forestland and cropland soils estimated using the ^15^N tracing model. Abbreviations: *M*_*N*_ total N mineralization (**a**); *I*_*NH4*_ total NH_4_^+^ immobilization (**b**); *O*_*Nrec*_ heterotrophic nitrification (**c**); *O*_*NH4*_ autotrophic nitrification (**d**); *I*_*NO3*_ NO_3_^−^ immobilization (**e**); DNRA: dissimilatory NO_3_^−^ reduction to NH_4_^+^ (**f**); nitrification capacity: ratio of *O*_*NH4*_ to *M*_*N*_ (**g**); NO_3_^−^ retention capacity ratio of total NO_3_^−^ consumption rates (*I*_*NO3*_ + DNRA) to the total NO_3_^−^ production rates (*O*_*NH4*_ + *O*_*Nrec*_) (**h**); *FL* forestland, *CL* cropland. The different letters in each sub-figure indicate significant differences between different land use soils (*P* < 0.05). The bottom/top of the box denote 25th/75th percentiles. Whiskers denote 5th/95th percentiles. The squares denote the mean values, and the black lines denote the mid-values.

The gross N mineralization (*M*_*N*_) rates averaged 3.95 ± 0.51 mg N kg^−1^ day^−1^ and 4.84 ± 0.69 mg N kg^−1^ day^−1^ in the forestland and cropland soils, respectively, with no significant difference (Fig. 3a). No significant relationships were detected between the mineralization rates and the measured soil properties.

**Figure 4:**
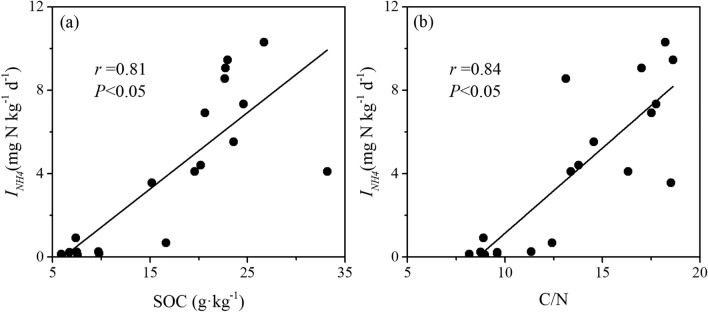
Relationships between soil gross NH_4_^+^ immobilization rates and SOC content (**a**) and C/N ratio (**b**).

The gross NH_4_^+^ immobilization (*I*_*NH4*_) rates in the cropland soils (0.11–0.92 mg N kg^−1^ day^−1^, mean 0.34 ± 0.10 mg N kg^−1^ day^−1^) were significantly lower than those in the forestland soils (3.56–10.31 mg N kg^−1^ day^−1^, mean 6.67 ± 0.74 mg N kg^−1^ day^−1^) (*P* < 0.05) (Fig. 3b). The soil gross NH_4_^+^ immobilization rates were positively related to the SOC content (*r* = 0.81) and the C/N ratio (*r* = 0.84) (Fig. 4).

**Figure 5:**
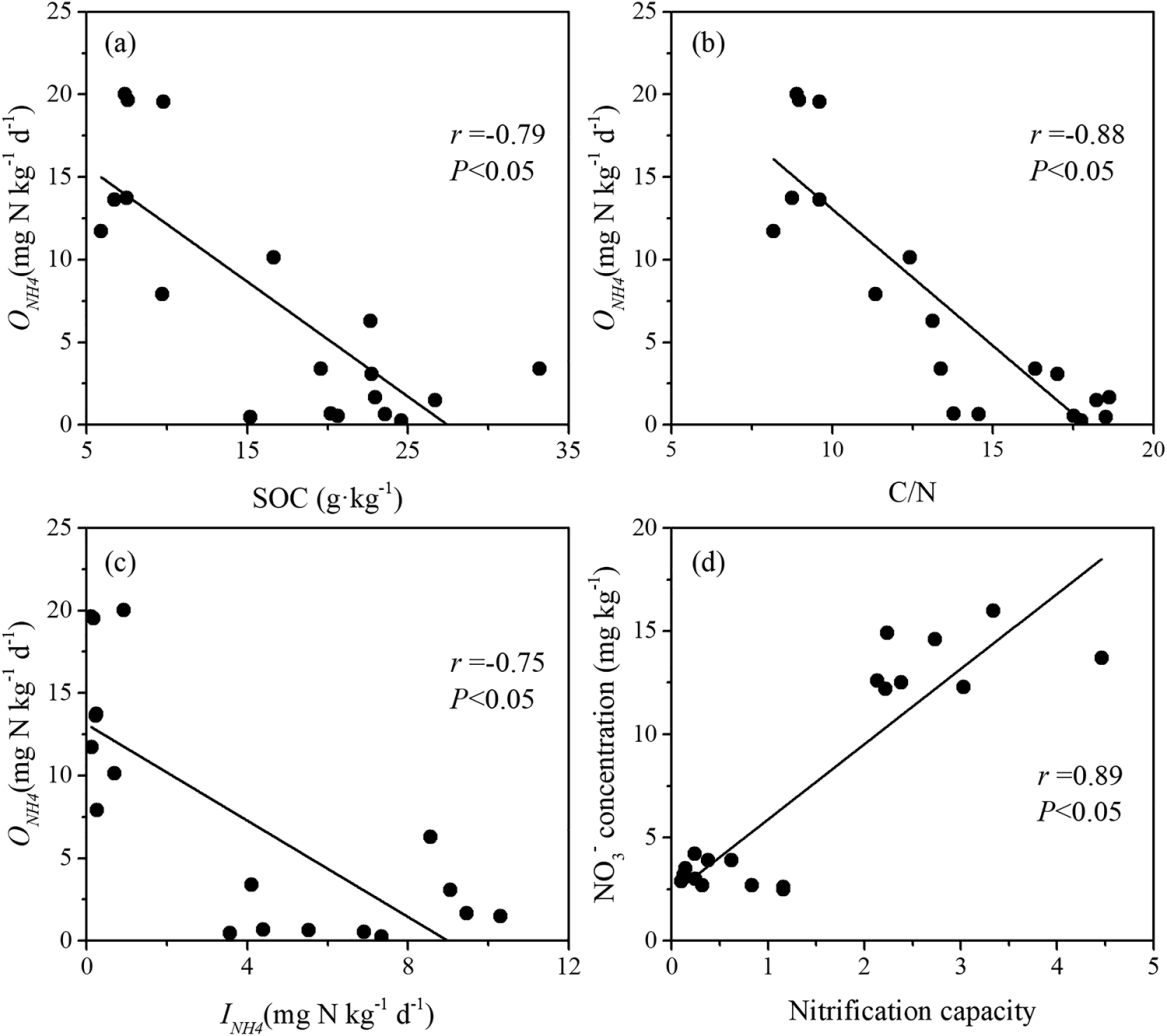
Relationships between soil gross nitrification rates and SOC content (**a**), C/N ratio (**b**) and gross NH_4_^+^ immobilization rates (**c**), and between nitrification capacity and NO_3_^−^ concentration (**d**). Nitrification capacity: ratio of *O*_*NH4*_ to *M*_*N*_.

Significantly higher gross autotrophic nitrification (*O*_*NH4*_) rates occurred in the cropland soils (7.91–20.02 mg N kg^−1^ day^−1^, mean 14.54 ± 1.66 mg N kg^−1^ day^−1^) than in the forestland soils (0.25–6.29 mg N kg^−1^ day^−1^, mean 1.99 ± 0.56 mg N kg^−1^ day^−1^) (*P* < 0.05) (Fig. 3d). However, gross heterotrophic nitrification (*O*_*Nrec*_) rates were negligible in both the forestland and cropland soils (Fig. 3c). The soil gross autotrophic nitrification rates were negatively related to the SOC content (*r* = − 0.79) (Fig. 5a) and the C/N ratio (*r* = − 0.88) (Fig. 5b). Negative correlations also existed between soil gross NH_4_^+^ immobilization and autotrophic nitrification rates (*r* = − 0.75) (Fig. 5c). Nitrification capacity (i.e., *O*_*NH4*_*/M*_*N*_) in the forestland soils (mean 0.48 ± 0.12) was lower than that in the cropland soils (mean 3.94 ± 1.23) (*P* < 0.05) (Fig. 3g). A positive correlation was detected between the NO_3_^−^ concentration and nitrification capacity (*r* = 0.89) (Fig. 5d).

The gross NO_3_^−^ immobilization (*I*_*NO3*_) and DNRA rates were not significantly different between the forestland and cropland soils (Fig. 3e,f). The cropland soils had a significantly lower NO_3_^−^ retention capacity (mean value of 0.01 ± 0.01) than the forestland soils (mean value of 0.51 ± 0.24) in this study (Fig. 3h).

### Soil NO_3_^−^ loss

**Figure 6:**
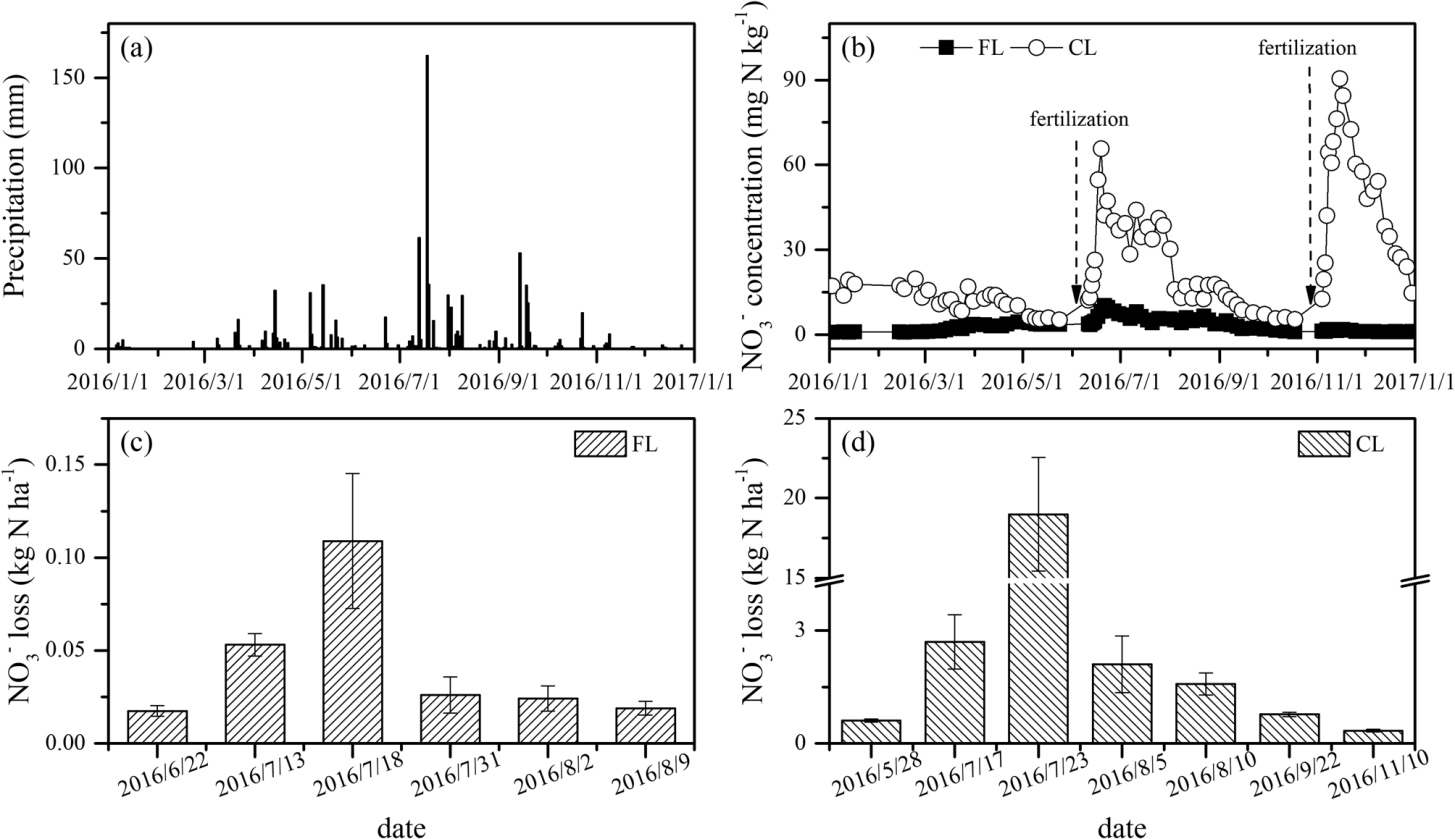
Dynamics of daily precipitation (**a**) and soil NO_3_^−^ concentration (**b**), and soil NO_3_^−^ losses at each runoff event from forestland (**c**) and cropland (**d**) during 2016. *FL* forestland, *CL* cropland.

During the whole year of 2016, six and seven runoff events were monitored in forestland and cropland sites, respectively (Fig. 6c,d). As shown, most NO_3_^−^ losses mainly occurred in summer season (from July to August), during which the rainfall was usually heavy and contributed 51% of the annual precipitation (Fig. 6a). In particular, the heaviest rainfall event was observed at 2016/7/18 with precipitation of 162 mm (Fig. 6a). Then, the highest NO_3_^−^ losses were monitored at 2016/7/18 in forestland and 2016/7/23 in cropland, respectively (Fig. 6c,d). The NO_3_^−^ losses in each runoff event were all significantly higher in cropland than in forestland (*P* < 0.05). Significantly higher soil NO_3_^−^ concentrations were also observed in cropland than that in forestland (*P* < 0.05), especially after the period of fertilization (Fig. 6b). The total NO_3_^−^ losses were 0.25 ± 0.01 kg N ha^−1^ year^−1^ and 27.10 ± 2.54 kg N ha^−1^ year^−1^ for the forestland and cropland, respectively.

## Discussion

### Patterns of gross N transformations in Regosols

Different with the generally acidic and highly weathered soils in humid subtropical regions^29^, the studied Regosols inherit most properties of parent materials and characterize by coarse texture and a neutral or alkaline reaction^18,22,23^. The specific soil properties of Regosols therefore may cause different N transformation processes compared to the normally occurring subtropical acidic soils.

In current study, gross N mineralization rates in the forestland soils (mean 3.95 ± 0.51 mg N kg^−1^ day^−1^) were similar to those observed in subtropical zonal soils of Orthic Acrisols and Humic Planosols (FAO soil classification) (mean 3.67 and 3.52 mg N kg^−1^ day^−1^, respectively)^4,34^. But they were higher than those measured in subtropical acidic Regosols of southwest China (mean 1.23 mg N kg^−1^ day^−1^)^35^. This difference is most likely related to the differences in soil organic matter and pH between these two Regosols^13,14^. Moreover, the measured gross NH_4_^+^ immobilization rates in the forestland soils (mean 6.72 ± 0.74 mg N kg^−1^ day^−1^) were higher than those observed in above mentioned subtropical soil types (0.82 mg N kg^−1^ day^−1^ to 2.25 mg N kg^−1^ day^−1^)^4,34,35^. These results indicated that the alkaline Regosols might have a faster NH_4_^+^ mineralization-immobilization turnover than other soil types in the subtropical regions.

The average total nitrification rates in forestland soils were 2.00 ± 0.57 mg N kg^−1^ day^−1^ in this study, and autotrophic nitrification contributed approximately 99.6%. This result indicated that autotrophic nitrification was the dominated NO_3_^−^ production process in the studied Regosols. However, significantly lower total nitrification rates were reported in subtropical acid soils, and heterotrophic nitrification was their dominant process^4,34,35^. Previous studies have showed that the microbiological autotrophic nitrification would be inhibited in soil at pH values lower than 5^36^, but be stimulated at pH values higher than 7.5^37^. Moreover, high soil pH might also inhibit the existence of fungi and their activities, which were related to heterotrophic nitrification^7^. Therefore, the differences in nitrification processes were likely related to the differences in soil pH between the alkaline Regosols and subtropical acid soils. Furthermore, the nitrification capacity (i.e., *O*_*NH4*_*/M*_*N*_ ratio, mean 0.48 ± 0.12) in present forestland soils was significantly greater than that in acidic Regosols (0.02) and Orthic Acrisols (0.05)^4,35^, which may therefore promote soil NO_3_^−^ accumulation and leaching risk in the study region^22–24^. This result could be verified by the high NO_3_^−^/NH_4_^+^ ratio in forestland soils in this study.

Gross NO_3_^−^ immobilization and DNRA were the important NO_3_^−^ consumption and retaining processes in soils^38,39^. DNRA generally occurred in anaerobic conditions^40–42^, however, it was negligible in this study due to the good aeration of Regosols. Gross NO_3_^−^ immobilization rates in this study were also significantly lower than those in other subtropical soils^4,34^. Previous studies have indicated that NO_3_^−^ immobilization generally needed high carbon availability^43^. The forestland soils in this study had lower soil organic C content (22.91 ± 1.37 g kg^−1^) compared to those subtropical forest soils^4,34^, which thus likely resulted the lower NO_3_^−^ immobilization. In addition, the inhibition of fungal activities by the high soil pH might also control the NO_3_^−^ immobilization in the studied Regosols^44^. Consequently, NO_3_^−^ retention capacity was significantly lower in alkaline Regosols (mean 0.51 ± 0.24) than that in subtropical zonal soils of Orthic Acrisols and Humic Planosols (0.98 and 0.81, respectively) under forestland^4,34^.

As discussed above, due to the specific soil properties, the non-zonal Regosols in the Sichuan Basin of Southwest China showed greatly different N transformation processes compared to the normally occurring soils in other subtropical regions. Overall, the alkaline Regosols had a faster NH_4_^+^ mineralization-immobilization turnover, higher nitrification rates, and lower NO_3_^−^ immobilization rates compared to other reported subtropical soils.

### Gross N transformations under different land use soils

The different land uses significantly affected the NH_4_^+^ immobilization and autotrophic nitrification in Regosols, while no significant differences were observed in gross mineralization, NO_3_^−^ immobilization and DNRA between the forestland and cropland soils (Fig. 3). In the cropland soils, the gross NH_4_^+^ immobilization rates were significantly lower than those in the forestland soils (Fig. 3b). This result agrees with most previous findings obtained from other subtropical soils in China^9,45^. However, Zhang et al. observed similar gross NH_4_^+^ immobilization rates between forestland and agricultural soils^4^. Soil gross NH_4_^+^ immobilization rates were positively related to the SOC and C/N in this study (Fig. 4). Previous studies indicated that relatively higher SOC content and C/N ratio in the soils could stimulate the increase of N immobilization potentiality^9,46^. In the cropland soils, due to long-term mineral N fertilizer application and very few crop residual retention, organic matter sources are mainly dependent on crop roots^9^. Therefore, the SOC content and C/N ratio in the cropland soils significantly decreased compared to those in the forestland soils (Fig. 2c,d), which might be an important factor that significantly reduced NH_4_^+^ immobilization. Furthermore, the gross NH_4_^+^ immobilization rates in the forestland soils were greater than the gross mineralization rates (Fig. 3a,b), indicating that a large proportion of the NH_4_^+^ produced from mineralization could be effectively immobilized. This rapid NH_4_^+^ turnover in forestland soils likely reduced the accumulation of NH_4_^+^, thus left little available substrate for nitrifiers^19,25^. However, in the cropland soils, the gross mineralization rates were significantly greater than the NH_4_^+^ immobilization rates (Fig. 3a,b), which might leave more available NH_4_^+^ substrates in soils for autotrophic nitrification.

The gross autotrophic nitrification rates were significantly greater in the cropland soils than in the forestland soils (Fig. 3d). This finding is in accordance with the results of some previous studies^4,9^. Soil pH is generally viewed as the key factor influencing nitrification^4,12,13^. However, soil pH was not significantly different between the forestland and cropland in this study (Fig. 2a). Nitrogen fertilization has been considered as another important factor stimulating nitrification in the cropland soils^4,9^. Numerous studies have indicated that long-term N fertilizer applications could stimulate autotrophic nitrification rates^18,30,47,48^. Applying mineral N fertilizer could induce the rapid increases in NH_4_^+^ concentrations for several weeks in cropland soils, thus providing sufficient available substrates for nitrification^49–51^. However, in this study, soil samples were collected once in April when several months had passed since the last fertilizer application. Thus, the extremely high nitrification rates following fertilization might not been considered in this study. Previous studies have shown that long-term N fertilizer application could affect the ammonia-oxidizing microbe population size and activity, thus stimulate autotrophic nitrification^18,30^. Previous studies in the same study area have revealed that application of mineral N fertilizer significantly increased soil ammonia-oxidizing bacteria (AOB) population size and changed AOB composition^49,50^. Consequently, long-term applying mineral N fertilizer might be the main factor inducing the differences in nitrification rates between the different land use soils in this study.

The ratio of soil gross autotrophic nitrification to gross NH_4_^+^ immobilization (*O*_*NH4*_/*I*_*NH4*_) can effectively indicate their relative importance in NH_4_^+^ consumption^5,9,10^. In this study, a negative correlation was observed between the gross autotrophic nitrification rates and NH_4_^+^ immobilization rates (Fig. 5c). In the forestland soils, the average *O*_*NH4*_/*I*_*NH4*_ ratio was 0.32 ± 0.09, indicating that NH_4_^+^ immobilization dominated NH_4_^+^ consumption. Conversely, the average *O*_*NH4*_/*I*_*NH4*_ ratio was 69.99 ± 18.91 in the cropland soils, indicating that autotrophic nitrification was the dominant NH_4_^+^-consuming process. These results also implied that autotrophic nitrification was greatly enhanced in the cropland soils than in the forestland soils in the study region.

Overall, in comparison to the forestland soils, gross autotrophic nitrification rates were significantly increased, while gross NH_4_^+^ immobilization rates were significantly decreased in the cropland soils. The significant differences in soil N transformations were closely related to the long-term mineral N fertilizer application, and the significant SOC content and C/N ratio decreases in the cropland soils.

### NO_3_^−^ loss and retention driven by soil N transformations

During the whole monitoring period, the cropland soils had significantly higher NO_3_^−^ concentrations than the forestland soils (Fig. 6b). The large amounts of NO_3_^−^ accumulation in the cropland soils could be easily diluted and lost during the heavy rainfall events^22–24^. The field monitoring results showed that the NO_3_^−^ losses in each runoff event were all significantly greater in the cropland soils than those in forestland soils (Fig. 6c,d). Previous studies indicated that the inorganic N form and amount in soils, especially the NO_3_^−^ accumulation, were controlled by N transformation processes^25,26^. In the cropland soils, the gross NH_4_^+^ immobilization significantly decreased but the gross autotrophic nitrification significantly increased compared to the forestland soils, resulting in the NO_3_^−^/NH_4_^+^ ratio (mean 5.79 ± 0.16) being 4 times greater than that in the forestland soils (mean 1.24 ± 0.05). This result confirmed that NO_3_^−^ not only was the dominant inorganic N form, but also has a higher concentration in the cropland soils.

The nitrification capacity (i.e., *O*_*NH4*_*/M*_*N*_ ratio) and *O*_*NH4*_/*I*_*NH4*_ ratio were two key indicators for the NO_3_^−^ loss potential from the soils^52,53^. In this study, compared to the forestland soils, the cropland soils had much higher nitrification capacity and *O*_*NH4*_/*I*_*NH4*_ ratio. The correlation analysis showed that the nitrification capacity and *O*_*NH4*_/*I*_*NH4*_ ratio were positively correlated with the NO_3_^−^ concentration and NO_3_^−^/NH_4_^+^ ratio (*P* < 0.05; Fig. 5d). Moreover, the low NO_3_^−^ retention capacity was observed in both the forestland and cropland soils (mean 0.51 ± 0.24 and 0.01 ± 0.01). Overall, the NO_3_^−^ production rates were greater than the NO_3_^−^ consumption rates, resulting in huge NO_3_^−^ accumulation in Regosols (mean 3.02 ± 0.18 mg N kg^−1^ and 13.60 ± 0.51 mg N kg^−1^ for forestland and cropland), which thus caused great risks of NO_3_^−^ losses, especially from the cropland soils^9,18,54^.

NO_3_^−^ losses caused nutrient loss and threatened the environment and human health^1,55,56^. In this study, the NO_3_^−^ losses occurring in the cropland soils were approximately 10% of the annual N fertilization. Consequently, NO_3_^−^ losses to the environment should be minimized by retaining NO_3_^−^ efficiently in the soils. As discussed above, the SOC content and C/N ratio significantly influenced soil N immobilization and nitrification. Thus, increasing the SOC content and C/N ratio would be effective strategies for NO_3_^−^ retention in the alkaline Regosols, which can potentially reduce autotrophic nitrification and enhance NH_4_^+^ immobilization.

## Conclusions

Compared to the typical zonal acidic soils in the subtropical regions, the non-zonal soils of alkaline Regosols in this study showed specifically inherent gross N transformations, i.e. the faster NH_4_^+^ mineralization-immobilization turnover, the higher nitrification rates, and the lower NO_3_^−^ immobilization rates. Different land use significantly affected the gross N transformation processes of autotrophic nitrification and NH_4_^+^ immobilization in Regosols. In the cropland soils, the rates of gross autotrophic nitrification were significantly greater, but the rates of gross NH_4_^+^ immobilization were significantly lower than those in the forestland soils. The specific soil gross N transformations resulted in low NO_3_^−^ retention capacity and thus high NO_3_^−^ loss risks in the Regosol croplands. The total NO_3_^−^ losses from the cropland soils were substantial (27.10 ± 2.54 kg N ha^−1^ year^−1^) and much greater than those from the forestland soils (0.25 ± 0.01 kg N ha^−1^ year^−1^). The great differences in the N transformations between the different land use soils may be attributed to the changes of the SOC content and C/N ratio and the application of mineral N fertilizer after long-term cultivation in the cropland.

## References

[1] Galloway JN (2008). Transformation of the nitrogen cycle: recent trends, questions, and potential solutions. Science.

[2] Zhu TB, Zhang JB, Cai ZC, Müller C (2011). The N transformation mechanisms for rapid nitrate accumulation in soils under intensive vegetable cultivation. J Soil Sediment..

[3] Zhang JB (2016). Soil gross nitrogen transformations along the Northeast China Transect (NECT) and their response to simulated rainfall events. Sci. Rep..

[4] Zhang JB (2013). Agricultural land use affects nitrate production and conservation in humid subtropical soils in China. Soil Biol. Biochem..

[5] Zhang YS (2018). Soil N transformation mechanisms can effectively conserve N in soil under saturated conditions compared to unsaturated conditions in subtropical China. Biol. Fert. Soil..

[6] Zhang JB, Cai ZC, Müller C (2018). Terrestrial N cycling associated with climate and plant-specific N preferences: a review. Eur. J. Soil Sci..

[7] Högberg MN, Högberg P, Myrold DD (2007). Is microbial community composition in boreal forest soils determined by pH, C-to-N ratio, the trees, or all three?. Oecologia.

[8] Rousk J (2010). Soil bacterial and fungal communities across a pH gradient in an arable soil. ISME J..

[9] Xu YB, Xu ZH (2015). Effects of land use change on soil gross nitrogen transformation rates in subtropical acid soils of Southwest China. Environ. Sci. Pollut. Res..

[10] Lang M, Li P, Han XZ, Qiao YF, Miao SJ (2016). Gross nitrogen transformations in black soil under different land uses and management systems. Biol. Fert. Soil..

[11] Yang WH, Ryals RA, Cusack DF, Silver WL (2017). Cross-biome assessment of gross soil nitrogen cycling in California ecosystems. Soil Biol. Biochem..

[12] Jiang XJ (2015). pH regulates key players of nitrification in paddy soils. Soil Biol. Biochem..

[13] Zhao W, Zhang JB, Müller C, Cai ZC (2018). Effects of pH and mineralisation on nitrification in a subtropical acid forest soil. Soil Res..

[14] Booth MS, Stark JM, Rastetter E (2005). Controls on nitrogen cycling in terrestrial ecosystems: a synthetic analysis of literature data. Ecol. Monogr..

[15] Osterholz WR, Rinot O, Liebman M, Castellano MJ (2017). Can mineralization of soil organic nitrogen meet maize nitrogen demand?. Plant Soil.

[16] Xu ZH (2008). Soil carbon and nutrient pools, microbial properties and gross nitrogen transformations in adjacent natural forest and hoop pine plantations of subtropical Australia. J. Soil Sediment..

[17] Yang LL (2008). Conversion of natural ecosystems to cropland increases the soil net nitrogen mineralization and nitrification in Tibet. Pedosphere.

[18] Wang J, Zhu B, Zhang JB, Müller C, Cai ZC (2015). Mechanisms of soil N dynamic s following long-term application of organic fertilizers to subtropical rain-fed purple soil in China. Soil Biol. Biochem..

[19] Burton J, Chen CG, Xu ZH, Ghadiri H (2007). Gross nitrogen transformations in adjacent native and plantation forests of subtropical Australia. Soil Biol. Biochem..

[20] Li DJ, Liu J, Chen H, Zheng L, Wang KL (2018). Soil gross nitrogen transformations in responses to land use conversion in a subtropical karst region. J. Environ. Manage..

[21] Zhang YS (2019). Land-use type affects nitrate production and consumption pathways in subtropical acidic soils. Geoderma.

[22] Wang T, Zhu B (2011). Nitrate loss via overland flow and inter flow from a sloped farmland in the hilly area of purple soil China. Nutr. Cycl. Agroecosys..

[23] Zhu B (2009). Measurements of nitrate leaching from a hillslope cropland in the central Sichuan basin China. Soil Sci. Soc. Am. J..

[24] Zhou MH (2012). Nitrate leaching, direct and indirect nitrous oxide fluxes from sloping cropland in the purple soil area, southwestern China. Environ. Pollut..

[25] Huygens D (2007). Soil nitrogen conservation mechanisms in a pristine south Chilean *Nothofagu*s forest ecosystem. Soil Biol. Biochem..

[26] Zhang JB (2016). The characteristics of soil N transformations regulate the composition of hydrologic N export from terrestrial ecosystem. J Geophys. Res-Biogeosci..

[27] Wang XG, Zhou MH, Li T, Ke Y, Zhu B (2017). Land use change effects on ecosystem carbon budget in the Sichuan Basin of Southwest China: Conversion of cropland to forest ecosystem. Sci. Total Environ..

[28] FAO/UNESCO. Soil map of the world. Revised legend. World Soil Resources Report No. 60, FAO, Rome, (1990).

[29] Müller C, Stevens RJ, Laughlin RJ (2004). A ^15^N tracing model to analyse N transformations in old grassland soil. Soil Biol. Biochem..

[30] Müller C, Rütting T, Kattge J, Laughlin RJ, Stevens RJ (2007). Estimation of parameters in complex ^15^N tracing models via Monte Carlo sampling. Soil Biol. Biochem..

[31] Zhang JB, Zhu TB, Cai ZC, Müller C (2011). Nitrogen cycling in forest soils across climate gradients in Eastern China. Plant Soil.

[32] Zhang JB, Zhu TB, Cai ZC, Qin SW, Müller C (2012). Effects of long-term repeated mineral and organic fertilizer applications on soil nitrogen transformations. Eur. J Soil Sci..

[33] Lu, R. K. *Soil Agro-chemical Analyses*. Agricultural Technical Press of China. (2000) (in Chinese).

[34] Zhu TB (2013). Nitrogen mineralization, immobilization turnover, heterotrophic nitrification, and microbial groups in acid forest soils of subtropical China. Biol. Fert. Soil..

[35] Wang J, Cheng Y, Zhang JB, Müller C, Cai ZC (2016). Soil gross nitrogen transformations along a secondary succession transect in the north subtropical forest ecosystem of southwest China. Geoderma.

[36] Weber DF, Gainey PL (1962). Relative sensitivity of nitrifying organisms to hydrogen ions in soils and solutions. Soil Sci..

[37] Wan YJ (2009). Gross nitrogen transformations and related nitrous oxide emissions in an intensively used calcareous soil. Soil Sci. Soc. Am. J..

[38] Chen ZM (2015). Importance of heterotrophic nitrification and dissimilatory nitrate reduction to ammonium in a cropland soil: Evidences from a ^15^N tracing study to literature synthesis. Soil Biol. Biochem..

[39] Zhang JB, Lan T, Müller C, Cai ZC (2015). Dissimilatory nitrate reduction to ammonium (DNRA) plays an important role in soil nitrogen conservation in neutral and alkaline but not acidic rice soil. J. Soil Sedim..

[40] Kraft B (2014). The environmental controls that govern the end product of bacterial nitrate respiration. Science.

[41] Pett-Ridge J, Silver WL, Firestone MK (2006). Redox fluctuations frame microbial community impacts on N-cycling rates in a humid tropical forest soil. Biogeochemistry.

[42] Rütting T, Boeckx P, Müller C, Klemedtsson L (2011). Assessment of the importance of dissimilatory nitrate reduction to ammonium for the terrestrial nitrogen cycle. Biogeosciences.

[43] Bradley RL (2001). An alternative explanation for the post-disturbance NO_3_^−^ flush in some forest ecosystems. Ecol. Lett..

[44] Jackson LE, Burger M, Cavagnaro TR (2008). Roots nitrogen transformations, and ecosystem services. Annu. Rev. Plant Biol..

[45] Xie Y (2018). Rapid recovery of nitrogen retention capacity in a subtropical acidic soil following afforestation. Soil Biol. Biochem..

[46] Schimel JP, Bennett J (2004). Nitrogen mineralization: Challenges of a changing paradigm. Ecology.

[47] Wang J (2017). Effects of 14 years of repeated pig manure application on gross nitrogen transformation in an upland red soil in China. Plant Soil.

[48] Dai SY, Wang J, Cheng Y, Zhang JB, Cai ZC (2017). Effects of long-term fertilization on soil gross N transformation rates and their implications. J Integr. Agr..

[49] Dong ZX, Zhu B, Hua KK, Jiang Y (2015). Linkage of N_2_O emissions to the abundance of soil ammonia oxidizers and denitrifiers in purple soil under long-term fertilization. Soil Sci. Plant Nutr..

[50] Dong ZX (2018). Seasonal N_2_O emissions respond differently to environmental and microbial factors after fertilization in wheat–maize agroecosystem. Nutr. Cycl. Agroecosys..

[51] Ren X, Zhu B, Bah H, Raza ST (2020). How tillage and fertilization influence soil N_2_O emissions after forestland conversion to cropland. Sustainability.

[52] Stockdale E, Hatch D, Murphy D, Ledgard S, Watson C (2002). Verifying the nitrification to immobilisation ratio (N/I) as a key determinant of potential nitrate loss in grassland and arable soils. Agronomie.

[53] Fernández-Fernández M, Rütting T, González-Prieto S (2017). Effects of a high-severity wildfire and post-fire straw mulching on gross nitrogen dynamics in Mediterranean shrubland soil. Geoderma.

[54] Zhao Y, Zhang JB, Müller C, Cai ZC (2018). Temporal variations of crop residue effects on soil N transformation depend on soil properties as well as residue qualities. Biol. Fert. Soil..

[55] Padilla FM, Gallardo M, Manzano-Agugliaro F (2018). Global trends in nitrate leaching research in the 1960–2017 period. Sci. Total Environ..

[56] Watanabe M (2018). Coniferous coverage as well as catchment steepness influences local stream nitrate concentrations within a nitrogen-saturated forest in central Japan. Sci. Total Environ..

